# Prevalence of hepatitis D virus in hepatitis B virus infected patients referred to Taleghani hospital, Tehran, Iran

**Published:** 2014

**Authors:** Seyed Mohammad Ebrahim Tahaei, Seyed Reza Mohebbi, Pedram Azimzadeh, Abbas Behelgardi, Azar Sanati, Parvaneh Mohammadi, Mahsa Khanyaghma, Armin Hosseini Razavi, Afsaneh Sharifian, Mohammad Reza Zali

**Affiliations:** 1 Gastroenterology and liver diseases Research center, Shahid Beheshti University of Medical Sciences, Tehran, Iran.; 2 Basic and Molecular Epidemiology of Gastrointestinal Disorders Research Center, Shahid Beheshti University of Medical Sciences, Tehran, Iran

**Keywords:** Prevalence, Hepatitis B virus, Hepatitis D virus, Iran

## Abstract

**Aim**: The aim of this study was to determine the prevalence of HDV infection between HBV chronic patients referred to gastroenterology ward of Taleghani hospital Tehran, Iran and also investigating the risk factors in acquiring the HDV infection.

**Background**: Hepatitis B virus (HBV) and Hepatitis D virus (HDV) are major public health issues. Worldwide there are approximately 350 million individuals chronically infected with the HBV. A significant part of them, including 15 to 20 million coinfected with HDV. Hepatitis Delta virus is transferred mostly through blood and body fluids.

**Patients and Methods**: HBV and HDV infections were evaluated by Enzyme-linked immunosorbent assay (ELISA). Liver functional tests were assessed through auto analyzer. Patients were interviewed and data along the test results were entered into SPSS program. We used chi-square, independent t-test and logistic regression for statistical analysis.

**Results**: 278 (54.6%) patients of the study group were male and 231 (45.4%) were female and the mean age of patients was 40.03 ± 14.93. From 509 patients, 39(7.7%) had anti-HDV antibody. In a uni-variable analysis, age (p=0.001), periodontal procedures (p=0.015), endoscopy (p=0.024) and colonoscopy (p=0.012) were significantly related to HDV seropositivity. After adjustment by logistic regression, age remained the only significant factor in acquiring HDV infection.

**Conclusion**: We highly recommend the health care workers to strictly follow the disinfection protocols of medical instruments. Since HDV seroprevalence changes over time, regular epidemiological studies are necessary to monitor the epidemiological trend of infection.

## Introduction

Hepatitis Delta virus (HDV) is a small defective RNA virus which needs Hepatitis B virus (HBV) for completion of its replication cycle inside the host cells. Hepatitis B virus provides HDV with its surface antigen, hepatitis B surface antigen (HBsAg), which HDV needs for transmission between hosts. However, HDV has a unique antigen, HDV Antigen, which is located underneath the outer HBsAg layer and is tightly associated with HDV RNA genome. Infection with HDV and HBV viruses can occur in two ways: it can happen simultaneously, co-infection or HDV can infect people after initial HBV infection, super-infection (1). Hepatitis B virus infection is a worldwide health problem, which is estimated that has infected more than 3.5 billion people globally. More than 350 million individuals suffer from chronic infection with this virus (2). The prevalence of HBV infection is high in South-East Asia and Sub-Saharan Africa, where more than 8% of the population are HBsAg chronic carriers (3). About 1.5 million people in Iran have been infected with HBV(4).

The rate of HDV seroprevalence is different in distinct parts of the world. Out of the 350 million people chronically infected with HBV, almost 15 million people have serological evidence of exposure to HDV(5). The seroprevalence of HDV is not always in accordance with HBV seroprevalence. For example, 90 percent of HBV carriers are infected with both viruses in the Paciﬁc Islands, whereas the rates decline to 8% in Italy and 5% in Japan (6). HDV infection is endemic in Middle East, Central Africa, Horn of Africa, Mediterranean, Eastern Europe, Amazon Basin and parts of Asia (7). In Iran and neighboring countries, where the infection is endemic, the rate of HDV seroprevalence in different regions is diverse (8, 9) and sometimes even in the same area the rate is different in distinct groups (10). 

The transmission routes of HDV are similar to those of HBV and researchers have shown that very little inoculums are sufficient for transmission (11). These routes include blood transfusion, intravenous drug abuse, sexual contact and nosocomial infection. There are some evidences that infection could be transmitted between family members (12, 13). This mode of transmission is especially important in countries, where the prevalence of virus is high (14). On the other hand, the intravenous drug abuse is important in northern Europe and countries with low prevalence of HDV (15).

The epidemiological trend of this virus is diverse in different parts of the world. With the introduction of blood screening programs and HBV vaccination, the rate of HDV infection has declined. This study aimed to investigate HDV seroprevalence in chronic HBV patients attending the gastroenterology ward of Taleghani hospital, Tehran, Iran and the risk factors in acquiring the infection.

## Patients and Methods

This cross sectional study was performed on patients attending the gastroenterology ward from 2006 to 2012. Each HBV patient who attended the gastroenterology ward of Taleghani hospital Tehran, Iran during this time was given a code. We utilized SPSS program to randomly select 509 patients from 1255 chronic hepatitis B patients based on their given code. Patients with HCV infection and other liver diseases such as non-alcoholic steatohepatitis (NASH) were excluded from this study. After gaining patients’ written consent, they were enrolled in this study. Each patient’s demographic information and records of any risky behavior like intravenous drug abuse, dangerous sexual contacts, cupping, hemodialysis, blood transfusion, tattooing, needle stick injury, periodontal procedures and shared razor were taken. The study was approved by the Institutional Medical Ethics Committees of Gastroenterology and Liver Diseases Research Center, Shahid Beheshti University of Medical Sciences. All participants signed a written consent and anonymity was warranted. The patients were consulted by a trained medical doctor and were informed of their test results.

Each patient’s blood was collected and its serum was separated and tested for the level of functional liver enzymes, ALT and AST (Auto analyzer Liasys Germany). The rest of the serum was stored in -70 freezers for further serologic tests. For confirmation of HBV infection, ELISA technique designed for HBsAg and anti-HBcIgG antibodies (Diapro Italy) was used. In the next step, the infection of these patients with HDV was assessed using ELISA technique (Diapro Italy) for the presence of anti-HDV IgG antibody. 

The demographic data and information gathered through interviews along with the test results were entered and described and analyzed by SPSS 19 program. Continuous variables are represented here as mean ± standard deviation and other parameters as frequency and percentage. Pearson’s chi-square was performed to test for independence of discrete variables. t-test was used to compare means of some continuous variable between two independent groups. Those factors whose p-value was below 0.2 in uni-variable analysis were analyzed with logistic regression for multivariate analysis. A p-value of 0.05 or less was considered statistically significant and all reported p-values were two sided.

## Results

Five hundred and nine HBV positive patients were enrolled in this study, which their HBV seropositivity was confirmed through ELISA technique. Mean age of patients at the time of sampling was 40.03 ± 14.93 and they were between 18 and 81 years old. Among them, 278 (54.6%) were male and 232 (45.4%) were female. The mean age of men and women was 41.04 ± 15.62 and 38.80 ± 13.99, respectively and there was no significant difference between the mean age in regards to the gender (p=0.09). HDV antibody was seen in 39 (7.7%) of HBV positive patients. Mean age of patients with HDV antibody was 47.59 ± 12.51 and the mean age of those without HDV infection was 39.39 ± 14.95. The difference of mean age between HDV positive and HDV negative was statistically significant (p=0.001). Anti-HDV antibody was seen in 18 (7.9%) of female patients and 21 (7.5%) of males. Gender was not significantly related to HDV seropositivity status (p=0.920).

The average of ALT level was higher in HDV positive patients in comparison to HDV negative patients, 50.95 ± 51.42 compare to 38.18 ± 56.00, respectively. However, it was not significantly related to HDV status (p=0.181). In addition, mean level of AST in anti-HDV positive patients was 48.51 ± 45.18 in comparison to 34.96 ± 53.20 in anti-HDV negative patients. This difference was not significantly related to HDV seropositivity status (p=0.139). 

Since no patients had the history of hemodialysis and none of them declared any history of high-risk sexual behavior, we omitted these two risk factors from statistical analysis. Transfusion (p= 0.089), cupping (p=1.00), tattoo (p=1.00), needle stick injuries (p=0.293), blood splashing (p= 0.070) and sharing shaving razor (p=0.166) have no significant relationship with HDV seropositivity. In uni-variable analysis, periodontal procedures (p=0.015), endoscopy (p=0.024) and colonoscopy were significantly related to HDV seropositivity (p= 0.012). The results are shown in Table 1.

 After entering all of the factors such as age, periodontal procedures, endoscopy and colonoscopy that were shown to be statistically significant regarding HDV seropositivity in uni-variable analysis into logistic regression, only age remained as a statistically significant factor (p=0.017).

## Discussion

Several different reports have been presented various results of HDV seropositivity among Iranian patients. This survey was performed to estimate the seroprevalence of HDV infection between HBV chronic patients referred to gastroenterology ward of Taleghani hospital, Tehran, Iran and also investigate the associated risk factors. Frequency of HDV antibody in our study was 7.7 percent. In this study we showed that age was significantly related to anti-HDV antibody seropositivity status between HBV patients that were attending gastroenterology ward of Taleghani hospital. 

**Table 1:** Distribution of different riskfactors among Anti-HDV Ab seropositive and seronegative groups.

Factor	HDV Negative (%)	HDV positive (%)	p-value	Odds Ratio (Confidence Interval)
Gender			0.920	1.034 (0.537-1.991)
Male	257 (92.4)	21 (7.6)		
Female	213 (92.2)	18 (7.8)		
Transfusion			0.089	2.401 (0.87-6.635)
No	441 (92.8)	34 (7.2)		
Yes	27 (84.4)	5 (15.6)		
Cupping			1.0	1.019 (0.384-2.710)
No	409 (92.3)	34 (7.7)		
Yes	59 (92.2)	5 (7.8)		
Tattoo			1.0	0.943 (0.277-3.206)
No	430 (92.3)	36 (7.7)		
Yes	38 (92.7)	3 (7.3)		
Needle stick injuries			0.293	2.054 (0.453-9.524)
No	456 (92.5)	37 (7.5)		
Yes	12 (85.7)	2 (14.3)		
Blood splashing			0.070	3.817 (1.005-14.490)
No	458 (92.7)	36 (7.3)		
Yes	10 (76.9)	3 (23.1)		
Sharing shaving razor			0.166	2.007 (0.839-4.803)
No	422 (93)	32 (7)		
Yes	46 (86.8)	7 (13.2)		
Endoscopy			0.024*	3.222 (1.235-8.403)
No	443 (93.1)	33 (6.9)		
Yes	25 (80.6)	6 (19.4)		
Colonoscopy			0.012*	9.667 (2.083-44.860)
No	464 (92.8)	36 (7.2)		
Yes	4 (57.1)	3 (42.9)		
Periodontal procedures			0.015*	2.602 (1.170-5.784)
No	188 (95.9)	8 (4.1)		
Yes	280 (90)	31 (10)		

*Entered into logistic regression

AST level was higher in HDV seropositive patients which is in accordance with results of Taghavi and colleagues (16) and Hajiani and caolleagues (17). ALT level was also higher in patients; these observations are seen in Xu study (18) and could be due to the fact that HDV infection could aggravate the inflammation of liver. Age was found statistically significant in regards to HDV seropositivity, which was in accordance with the Hajiani study (17). However, in another study this association was not found (19). We did not find any relationship between gender and HDV seropositivity status that is in contradiction with Taghavi study (16), but in accordance with Hajiani and Ataei studies (17, 19). 

Rate of HDV seroprevalence differs in different countries. In Africa, in countries like Cameron and Nigeria the rate of HDV seropositivity reaches 17.6% (20) and 12.5 percent (21). The seroprevalence rates in India 5-10% (22), Thailand 0.7% (23) and Bangladesh 24.4% (24) shows diversity among Asian countries.

In northern Europe the rate of HDV seroprevalence in some regions is high. For example, HDV seroprevalence rate in Germany was 14% (25) while in Poland this rate was 4.8% (26). In neighboring countries of Iran, the rate of prevalence is very different; in Pakistan, Turkey and Saudi Arabia the seroprevalence rates were 16.6%, 5.2% and 3.3%, respectively (27, 28). 

In Iran, Rezvan and colleagues reported the rate of anti-HDV seropositivity in asymptomatic HBV carriers was 2.5 percent, while in dialysis patients who were HBsAg positive, this rate reaches 44.5 percent (29). This shows that these people are in greater risk of getting the infection than the rest of HBs positive patients. Alavian and colleagues in a study on patients attended the hepatitis center in Tehran, Iran, reported that serum antibody was present among 5.9% of HBV patients(30). 

Different studies in different parts of Iran show that HDV seroprevalence rate is diverse. Ataei and colleagues reported that the rate of HDV seroprevaelce in Isfahan province, central Iran is 2.9% (19). Taghavi and colleagues from Shiraz, southern Iran reported a high rate of 9.7% of anti-HDV seropositivity (16). However, Malekzadeh had reported a different rate of 13.9 percent from the same province, though in a different time frame (31). This observation shows that during this period of time, the rate of HDV seropositivity in this part of Iran had been decreasing. In Zahedan, a southeastern city of Iran, HDV seroprevalence was reported 17% by Bakhshipour and colleagues during a period of time from 2008 to 2011 (32). In Hamedan, Amini and colleagues reported a 2.4% of HDV seroprevalence in general population (10). However, Alizadeh and colleagues reported the seroprevalence of HDV 17.3% among HBsAg positive patients who had been referred to Hepatitis community center of Hamedan province. In southern shores of Caspian sea, Hassanjani reported the rate of 2% seropositivity (33). However, in southeastern shore of the Caspian sea, in Golestan province, Roshandel and colleagues reported a higher rate of 5.8% seropositivity (34). A recent survey from asymptomatic blood donors was performed by Attaran and colleagues, which revealed 2% seropositivity for anti-HDV (35).

These studies alongside our current study depict a different picture from the HDV seroprevalence trend in other parts of the world; while in other parts of the world the rate of HDV seropositivity has declined, by introducing HBV vaccine and screening the blood for blood borne infections (36-38), the rate of HDV seropositivity in Iran has increased. This epidemiological trend has been seen in some parts of the world as well (39-41), even in industrial countries like UK and Germany (25, 42, 43). 

In conclusion, we recommend that all of HBV patients should also be screened for HDV infection as well. In addition, we recommend that the disinfection of utensils become mandatory and screened by healthcare officials in dentist offices and endoscopy and colonoscopy wards.

## Acknowledgment

This work was supported by a grant from Gastroenterology and Liver Diseases Research Center, Shahid Beheshti University of Medical Sciences, Tehran, Iran.

## References

[1] Rizzetto M (2009). Hepatitis D: Thirty years after.. J Hepatol.

[2] de Franchis R, A H, G L, D L, A L, N M, al e (2002). EASL International Consensus Conference on Hepatitis B. 13-14 September, 2002 Geneva, Switzerland. Consensus statement (long version). J Hepatol.

[3] Ganem D, Prince A (2004). Hepatitis B virus infection--natural history and clinical consequences.. N Engl J Med.

[4] Hb A, M A, A K, K B (2008). Hepatitis B virus infection in Iran: A systematic review.. Hepat Mon.

[5] Farci P (2003). Delta hepatitis: An update.. J Hepatol.

[6] Rizzetto M, Ponzetto A, Forzani I (1991). Epidemiology of hepatitis delta virus: Overview.. Prog Clin Biol Res.

[7] Pa R, I F (1990). Hepatitis Delta virus as a global health problem.. Vaccine.

[8] Toukan A, e. Oa A, Sa A.-L, Ms T, Mf K, Sc H, al e (1987). The epidemiology and clinical outcome of hepatitis D virus $\delta$ infection in Jordan. Hepatology.

[9] Al-Kandari S, Nordenfelt E, Al-Nakib B, Hansson B, Ljunggren K, Al-Nakib W (1988). Hepatitis delta virus infection in acute hepatitis in Kuwait.. Scand J Infect Dis.

[10] Amini S, Mahmoodi M, Andalibi S, Solati A (1993). Seroepidemiology of hepatitis B, delta and human immunodeficiency virus infections in Hamadan province, Iran: A population based study.. J Trop Med Hyg.

[11] Ponzetto A, Hoyer B, Popper H, Engle R, Purcell R, Gerin J (1987). Titration of the infectivity of hepatitis D virus in chimpanzees.. J Infect Dis.

[12] Bonino F, N C, P D, G M, L V, A C, al e (1985). Familiar clustering and spreading of hepatitis delta virus infection.. J Hepatol.

[13] Niro G, Jl C, E G, M G, P C, E S, al e (1999). Intrafamilial transmission of hepatitis delta virus: Molecular evidence.. J Hepatol.

[14] Hughes S, Wedemeyer H, Harrison P (2011). Hepatitis delta virus. Lancet.

[15] Novick D, P F, Ts C, Mb T, Cw S, Me L, al e (1988). Hepatitis D virus and human immunodeficiency virus antibodies in parenteral drug abusers who are hepatitis B surface antigen positive.. J Infect Dis.

[16] Ss T, D M, F K (2008). Hepatitis D in Chronic Active Hepatitis B: Prevalence, Liver Enzyme Levels and Histopathology-an Epidemiological Study in Shiraz, Southern Iran, 2003-2004.. Hepat mon.

[17] Hs H, F J (2009). Seroprevalence of Delta Hepatitis in Patients with Chronic Hepatitis B and its Clinical Impact in Khuzestan Province, Southwest Iran.. Hepat mon.

[18] Gu X, Li Q, Wang Y (2001). Clinical characteristics of the patients with hepatitis B combining hepatitis D infection. Zhonghua Gan Zang Bing Za Zhi.

[19] Ataei B, Mr Y, H K, M Y, Z N, Aa J, al e (2011). Hepatitis D virus infection in Isfahan, central Iran: Prevalence and risk factors among chronic HBV infection cases. Hepat Mon.

[20] Foupouapouognigni Y, Noah D, Sartre M, Njouom R (2011). High prevalence and predominance of hepatitis delta virus genotype 1 infection in Cameroon. J. Clin. Microbiol..

[21] Nwokediuko S, Ijeoma U (2009). Seroprevalence of antibody to HDV in Nigerians with hepatitis B virus-related liver diseases.. Niger J Clin Pract.

[22] Acharya S, Madan K, Dattagupta S, Panda S (2006). Viral hepatitis in India.. Natl Med J India.

[23] Mk L, B S, C K, C K, P K (2002). The prevalence of viral hepatitis among the Hmong people of northern Thailand. Southeast Asian J Trop Med Public Health.

[24] Zaki H, Darmstadt G, Baten A, Ahsan C, Saha S (2003). Seroepidemiology of hepatitis B and delta virus infections in Bangladesh. J Trop Pediatr.

[25] Wedemeyer H, Heidrich B, Manns M (2007). Hepatitis D virus infection--not a vanishing disease in Europe!. Hepatology.

[26] Bielawski K, Zietkowski D, Charmuszko U, Sikorska K, Stalke P (2006). Hepatitis delta virus infection in chronically HBV-infected patients from northern Poland.. Arch. Virol.

[27] Mumtaz K, Ss H, S A, A A, M I, S A, al e (2005). Epidemiology and clinical pattern of hepatitis delta virus infection in Pakistan. J Gastroenterol Hepatol.

[28] As A (2005). Hepatitis D Virus Infection; Iran, Middle East and Central Asia. Hepat Mon.

[29] Rezvan H, Forouzandeh B, Taroyan S, Fadaiee S, Azordegan F (1990). A study on delta virus infection and its clinical impact in Iran. Infection.

[30] As A, H M, M M, T D, B H (2005). Frequency and risk factors of hepatitis D virus in hepatitis B patients. GOVARESH JOURNAL.

[31] Bf M (1989). Prevalence of HDV in asymptomatic HDV healthy carriers of HBV in Iran. Irn J Med Sci.

[32] Bakhshipour A, Mashhadi M, Mohammadi M, Nezam S (2013). Seroprevalence and risk factors of hepatitis delta virus in chronic hepatitis B virus infection in Zahedan. Acta Med Iran.

[33] Th H.-R (2002). Frequency of chronic active hepatitis in asymptomatic HBV carriers in Babol, Iran. Arch Iranian Med.

[34] Ss R, N A, S B, Aa K, H J (2008). Prevalence of hepatitis D virus infection in hepatitis B surface antigen-positive subjects in Golestan province, northeast Iran. J Microbiol Immunol Infect..

[35] Attaran M, Sharifi Z, Hosseini S, Samei S, Ataee Z (2014). Prevalence of hepatitis B and hepatitis D coinfection in asymptomatic blood donors in Iran. APMIS.

[36] Degertekin H, Yalcin K, Yakut M (2006). The prevalence of hepatitis delta virus infection in acute and chronic liver diseases in Turkey: An analysis of clinical studies. Turk J Gastroenterol.

[37] Navascues C, M R, Ng S, P S, A L, A S, al e (1995). Epidemiology of hepatitis D virus infection: Changes in the last 14 years. Am J Gastroenterol.

[38] Huo T, Wu J, Lin R, Sheng W, Chang F, Lee S (1997). Decreasing hepatitis D virus infection in Taiwan: An analysis of contributory factors. J Gastroenterol Hepatol.

[39] Chakraborty P, U K, A J, R G, Rk G, Bc D, al e (2005). Seroprevalence of hepatitis D virus in patients with hepatitis B virus-related liver diseases. Indian J Med Res.

[40] Seetlani N, Abbas Z, Raza S, Yakoob J, Jafris W (2009). Prevalence of hepatitis D in HBsAg positive patients visiting liver clinics. J Pak Med Assoc.

[41] Makuwa M, Mintsa-Ndong A, Souquiere S, Nkoghe D, Leroy E, Kazanji M (2009). Prevalence and molecular diversity of hepatitis B virus and hepatitis delta virus in urban and rural populations in northern Gabon in central Africa. J. Clin. Microbiol..

[42] Ag S, G W, T M, I M, R W (1992). Hepatitis B and delta virus infection among" at risk" populations in south east London. J Epidemiol Community Health.

[43] Cross T, P R, M H, A J, Mj H, Hm S, al e (2008). The increasing prevalence of hepatitis delta virus (HDV) infection in South London. J Med Virol.

